# Knowledge Structure and Emerging Trends of Mild Cognitive Impairment with Dyssomnias in Recent 20 Years: A Bibliometric Analysis via CiteSpace and VOSviewer

**DOI:** 10.1155/2024/6622212

**Published:** 2024-01-06

**Authors:** Haoyu Huang, Zesen Zhuang, Yiwen Wan, Jiao Shi, Xu Yuan, Dan Wang, Shangjie Chen

**Affiliations:** ^1^College of Rehabilitation Medicine, Fujian University of Traditional Chinese Medicine, Fuzhou 350122, China; ^2^Department of Rehabilitation Medicine, The Second Affiliated Hospital of Shenzhen University, Shenzhen 518101, China

## Abstract

**Background:**

Mild cognitive impairment (MCI), an intermediate stage between normal aging and dementia, has emerged as a prominent research area in geriatric care due to its heightened propensity for progressing toward dementia. Sleep plays a pivotal role in cognitive function, with dyssomnias not only exacerbating cognitive and affective symptoms associated with neurodegenerative diseases but also contributing to disease progression.

**Aim:**

This bibliometric analysis investigates the global research on MCI with dyssomnias over the past two decades, aiming to discern key findings, research domains, and emerging trends in this field.

**Methods:**

In this study, a bibliometric analysis was conducted using the search terms “MCI” and “sleep”. Data were extracted from the Web of Science Core Collection database, and visualization and collaborative analysis were performed using CiteSpace and VOSviewer.

**Results:**

This study encompassed 546 publications from 2003 to 2023. The publication volume and citation rate consistently increased over time. Neurosciences, Clinical Neurology, and Geriatrics Gerontology emerged as the top three research fields. The *Journal of Alzheimer's Disease* had the highest publication count, while *Sleep Medicine* received the most citations. USA, China, and Italy led in publication output. Collaborative clusters among authors and institutions were identified, but cooperation between clusters was limited. Active cocited reference clusters included “obstructive sleep apnea”, “possible mediating pathways”, and “isolated rapid eye movement sleep behaviour disorder”. The top frequently mentioned keywords, besides “MCI”, were “Alzheimer's disease”, “dementia”, “risk factor”, and “Parkinson's Disease”. Notable keyword clusters spanned circadian rhythm, Parkinson's disease, MCI, dementia with Lewy body, subjective cognitive impairment, Lewy body disease, Alzheimer's disease, and dietary patterns.

**Conclusion:**

The field of MCI with dyssomnias is rapidly expanding, encompassing a wide range of neurodegenerative disorders and sleep disturbances. Current research endeavors are primarily focused on elucidating the underlying pathogenesis, predicting disease progression, and developing innovative treatment strategies for individuals affected by MCI with dyssomnias.

## 1. Introduction

Mild cognitive impairment (MCI) is characterized by a subtle decline in cognitive abilities that surpasses normal age-related changes but does not significantly impact daily functioning. It serves as an intermediary stage between typical cognitive aging and dementia. Although individuals with MCI may experience difficulties in memory, attention, language, executive functions, or visuospatial skills, their impairment is not severe enough to warrant classification as dementia [1]. It is noteworthy that not all cases of MCI progress to dementia, some may stabilize or even revert to normal cognitive function over time. However, individuals with MCI are at a higher risk of developing dementia compared to those without it [2]. Various factors contribute to the development of MCI, including vascular issues, depression, anxiety, and sleep disorders [3, 4].

Quality sleep is essential for optimal cognitive health, as it supports attention, problem-solving abilities, creativity, and decision-making processes. During sleep, the brain undergoes crucial processes such as waste elimination, cellular repair, and energy replenishment that enable it to function at its best [5]. Dyssomnias, such as chronic sleep deprivation, fragmented sleep, and excessive daytime sleepiness, can significantly impact the quality of life and impair cognitive functions like memory and attention. Research suggests that sleep disorders not only exacerbate cognitive and affective symptoms associated with neurodegeneration but also contribute to disease progression [6]. Dyssomnias have been identified as a significant risk factor for MCI, with a prevalence of approximately 70.1% in individuals with MCI compared to around 56.5% in those who are cognitively healthy [7–9]. Furthermore, the severity of dyssomnias tends to be higher in individuals with MCI. MCI is frequently accompanied by sleep and circadian rhythm disorders, which significantly contribute to cognitive decline [10]. Different subtypes of MCI exhibit distinct patterns of sleep disorders, with patients diagnosed with amnestic mild cognitive impairment (aMCI) exhibiting a lower arousal index and those diagnosed with nonamnestic mild cognitive impairment (naMCI) showing longer total sleep time but also a lower arousal index [11]. Understanding the relationship between MCI and sleep disorders, exploring their underlying mechanisms, and developing effective interventions are crucial for optimizing outcomes in individuals with MCI and dyssomnias.

Scientometrics is a discipline that employs quantitative analysis of scientific publications, citations, and bibliometric data to comprehend various aspects of scientific research. It involves examining patterns, relationships, and trends within the scientific literature to gain insights into the structure and dynamics of scientific knowledge [12, 13]. However, bibliometric analyses of research on MCI with dyssomnias are currently lacking. In this study, we utilized CiteSpace 6.1.R6 (64 bits) and VOSviewer 1.6.18 for scientometric literature visualization and analysis to explore and identify the current research hotspots in this field over the past two decades, as well as predict future trends.

## 2. Materials and Methods

### 2.1. Data Sources and Search Strategy

**Figure 1:**
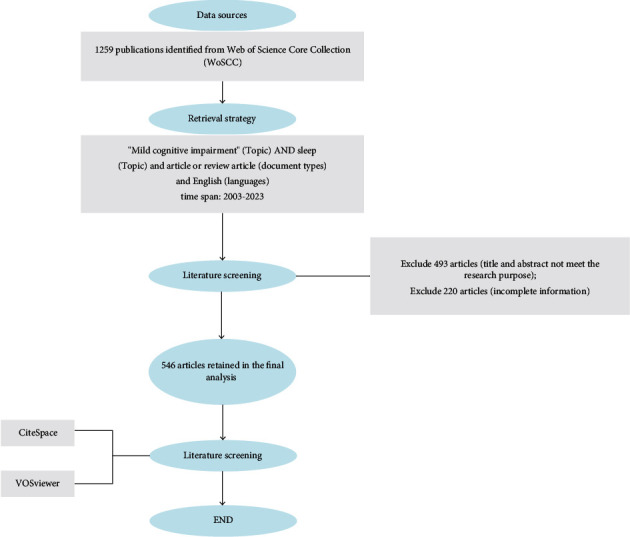
Flowchart for literature screening.

The Web of Science Core Collection (WoSCC) was utilized to retrieve studies pertaining to MCI with dyssomnia research from January 1, 2003, to April 30, 2023. To obtain relevant literature on MCI with dyssomnia research in the past two decades, we employed the following search strategy (see Figure 1): “mild cognitive impairment” (Topic) AND “sleep” (Topic) and Article or Review Article (Document Types) and English (Languages).

The WoS tool was utilized to convert all data into plain text format, which were subsequently subjected to further bibliometric analysis using CiteSpace 6.1.R6, 64-bit (Drexel University, Philadelphia, PA, USA) and VOSviewer 1.6.18 (Leiden University, Van Eck NJ).

### 2.2. Analysis Methods

We employ CiteSpace and VOSviewer to discern collaborations among countries/territories, institutions, and authors, as well as to identify keywords, journals, major cocited journals, and references, thereby constructing pertinent visualization networks. Both CiteSpace and VOSviewer facilitate researchers in visually exploring and analysing scientific literature by identifying clusters of interconnected research areas, citation networks, emerging trends, and collaborative efforts.

CiteSpace, developed by Chaomei Chen, is a software tool specifically designed for the visual analysis of citation networks in scientific literature [14]. It generates maps and timelines that highlight significant citation clusters, emerging trends, influential papers, and research frontiers. Each node represents individual items such as papers, authors, or research topics with node size indicating their importance or impact. The coloured circular ring of a node is called the tree ring history, representing the citation history of a specific article. The overall size of the ring reflects the number of times the paper has been cited, while its colour represents the corresponding time period in which it was cited. Additionally, the thickness of a ring is directly proportional to the number of citations within that specific time period. Relationships between items are depicted through connecting lines, with the thickness of the lines indicating citation strength or frequency. The centrality measures such as degree centrality and betweenness centrality can help identify influential nodes. The outermost purple circle represents node centrality, with thicker circles denoting higher levels of centrality. Higher centrality values indicate greater prominence or influence, revealing key papers, authors, or research topics with significant impact or serving as bridges between clusters, thus highlighting their crucial role in the scholarly network. The CiteSpace analysis image generation process utilized the default parameters. The time period from 2003 to 2023 was divided into 20 intervals for analysis, and the unit of time slicing is one year. The default selection was based on the g-index and a scaling factor *k* of 25 (*k* = 25). The link retaining factor (LRF) was set at 3, and the maximum links per node (L/N) parameter was set to 10. The look back years (LBY) parameter was set to 5. In the equation TopN = {n|f(n) > = e}, the value of *e* was set to 1. Additionally, we applied Pathfinder network scaling within the Pruning panel.

VOSviewer, a widely employed visualization tool for scientometric analyses [15], facilitates the generation of maps that visually represent relationships among various elements, such as authors, keywords, journals, or research institutions, based on patterns of cooccurrence or cocitation. The cooperation network diagram visualizes collaboration patterns. By analysing the cooperation network diagram, researchers can identify items, such as important journals, countries, or territories that have significant collaborative activity within the analysed literature.

## 3. Results

### 3.1. Annual Growth Trends and Citation Trends

**Figure 2:**
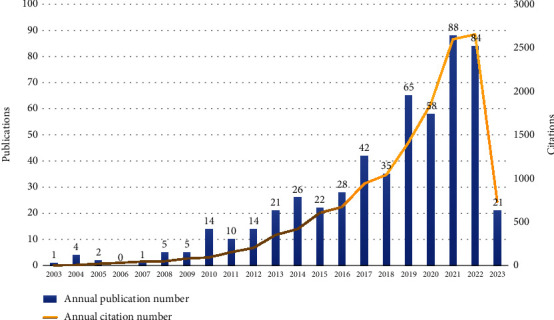
The annual number of publications and citations on MCI with dyssomnias has been recorded from 2003 to 2023.

A total of 546 articles related to MCI with dyssomnias were identified in the WoSCC database.  Figure 2 presents the publication and citation counts from 2003 to 2023, where publications are represented by blue bars and citations by a yellow line. The quantity of publications and citations within this particular field has exhibited a noticeable upward trend over the past two decades. The field of MCI with dyssomnias has attracted significant attention, resulting in a total of 13,957 citations with an average of 25.56 citations per paper. Initially, there were only a limited number of publications annually; however, since 2010, substantial growth has been observed, culminating in the highest number of publications in 2021. Although there was a slight decline in 2022, the citation count continues to rise.

### 3.2. Research Areas and Most Cited Articles

The research topic covered a wide range of 58 distinct research fields related to MCI with dyssomnias, with Neurosciences and Clinical Neurology occupying a prominent position, followed by Geriatrics Gerontology ranking third.

The top 10 most cited articles and their primary findings are presented in Table 1. Among these articles, six specifically highlight the correlation between sleep disorders and the susceptibility to neurodegenerative diseases. Additionally, the highly cited articles not only elucidated the underlying pathological mechanisms linking neuropsychiatric symptoms, such as sleep disorders, with neurodegenerative diseases but also encompassed comprehensive discussions on the screening, diagnosis, and treatment of sleep disorders associated with cognitive impairment in elderly individuals.

### 3.3. Analysis of Author and Coauthor Collaboration

**Figure 3:**
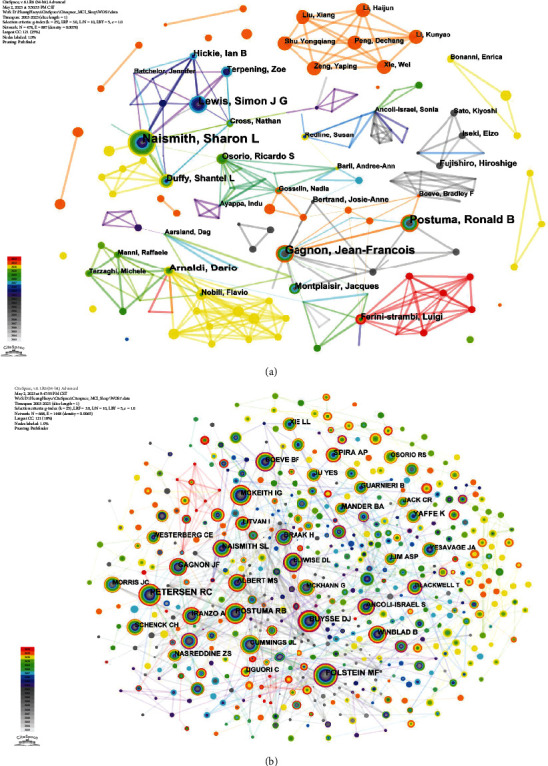
The visualization map displays participating authors (a) and cocited authors (b) for MCI with dyssomnias.

Between 2003 and 2023, a total of 3,466 authors contributed to research on MCI with dyssomnias. The node size and colour in the map correspond to the number of citations and a single time slice, respectively. The author and cocited author collaboration network map provide valuable insights into potential collaborators, facilitating the establishment of collaborative relationships among researchers (see Figures 3(a) and 3(b)). The cooperative network map constructed by the author revealed a significant number of researchers (478 nodes) and a strong collaborative relationship (887 lines) among them. Six major research teams have emerged in this field, including Sharon L Naismith, Simon J G Lewis, Postuma Ronald B, Gagnon Jean-Francois, Arnaldi Dario, and Luigi Ferini-Strambi. Each team demonstrates strong internal connections, fostering effective interteam collaboration. Among the cocited authors, Ronald C Petersen, Marshal F Folstein, and Ronald B Postuma emerge as the top three most frequently cited researchers. In terms of centrality measures, Donald L Bliwise, Sonia Ancoli-Israel, and Marshal F Folstein exhibit the highest values.

### 3.4. Major Research Institutions and Most Productive Countries/Territories

**Figure 4:**
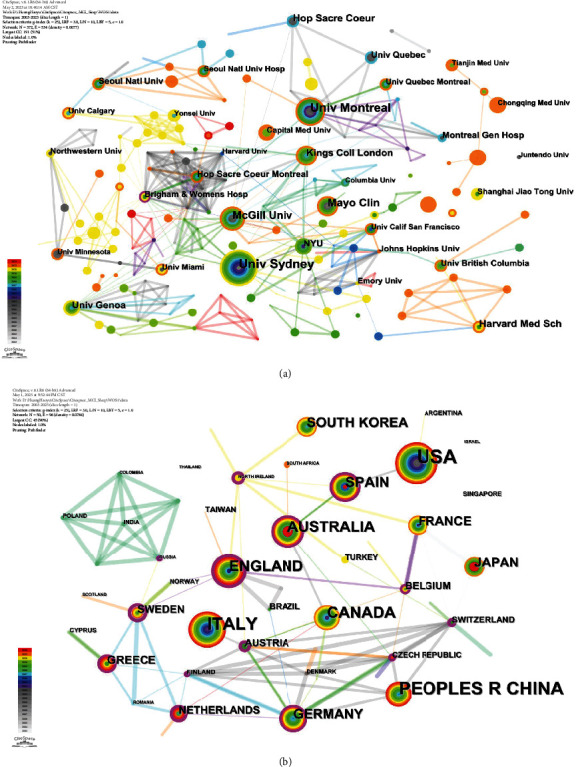
The visualization map displays institutions (a) and countries/territories (b).

A total of 2,520 institutions contributed articles on MCI with dyssomnias. Among them, the University of Sydney, Université de Montréal, and McGill University ranked as the top three institutions in terms of publication volume. Regarding citations, Université de Montréal, Mayo Clinic, and Hôpital du Sacré-Cœur de Montréal emerged as the leading institutions. Notably, the Université de Montréal exhibited exceptional productivity and citation impact, indicating a high-quality research output. Using CiteSpace, Figure 4(a) visualizes the network of institutions comprising 372 nodes and 534 lines. The figure reveals distinct collaborative clusters among regional institutions; however, the level of cooperation between these clusters is not extensive enough.

A total of 227 countries/territories have contributed to the literature on MCI with dyssomnias, demonstrating international collaboration in this field.  Figure 4(b) depicts the collaborative networks and publication density of each country/territory, while Table 2 highlights the top 10 most productive nations in terms of published literature. The United States ranks first in terms of publication volume, closely followed by China as the second-largest contributor, and Italy follows suit. In terms of centrality, Sweden exhibits the highest level, while the United Kingdom and Northern Ireland rank second and third, respectively. The top three countries in citation frequency are the United States, Canada, and Spain; these countries also demonstrate high levels of centrality and citation rates. Overall, these countries have played a significant role in the research field of MCI with dyssomnias and have made substantial contributions to this topic.

### 3.5. Analysis of Journal Collaboration and Volume of Publications

**Figure 5:**
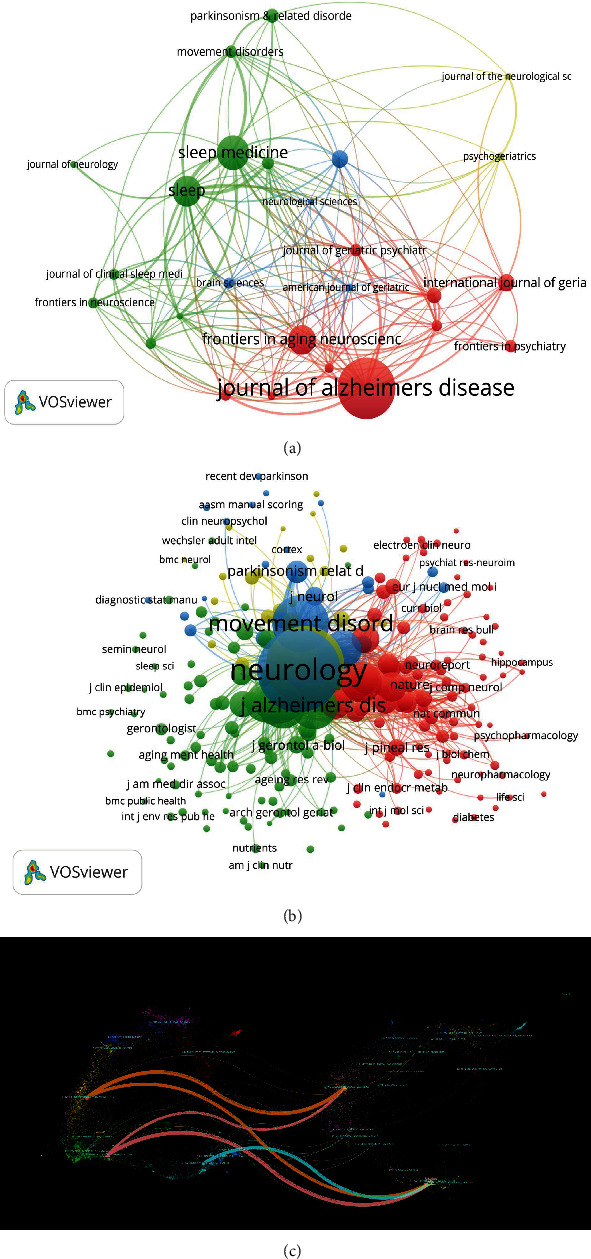
The visualization maps of citation networks for journals (a) and cocitation networks for journals (b), along with the dual-map overlay of journals (c).

The data analysis encompassed a total of 194 journals specializing in MCI with dyssomnias. Figures 5(a) and 5(b) depict the citation network and cocitation network maps, respectively, of these journals, which were generated using VOSviewer. In these visual representations, larger nodes indicate journals with higher publication volumes.  Table 3 provides an overview of the top 10 most cited journals and cocited journals based on both publication quantity and citation frequency. The *Journal of Alzheimer's Disease* exhibits the highest publication volume, while *Sleep Medicine* demonstrates the most frequent journal citation frequency, thereby emphasizing their paramount significance within the field. *Neurology*, *Sleep*, and *Movement Disorders* emerge as the top three cocited journals, thus endorsing their authoritative status as trusted sources in this domain. These esteemed journals hold substantial importance and wield significant influence, indicating that the literature they publish surpasses the average standard.

Figure 5(c) presents an overlay diagram of dual graphs generated by CiteSpace, depicting the distribution of journals pertaining to MCI with dyssomnias. This diagram facilitates researchers in comprehending the knowledge flow and frontier hotspots across diverse disciplines. The left side of the overlay atlas represents the fields of citing literature, while the right side signifies those of cited literature. The figure illustrates five primary reference paths. The left side corresponds to the citation map, while the right side represents the cited journal map. Molecular/biology/immunology journals are depicted by the orange path, Neurology/Sports/Ophthalmology journals are represented by the pink path, and Psychology/Education/Health journals are indicated by the blue path in relation to Psychology/Education/Social areas. Furthermore, both the orange and pink paths are also cited within the Molecular/Biology/Genetics domains.

### 3.6. Analysis of Cocited References

**Figure 6:**
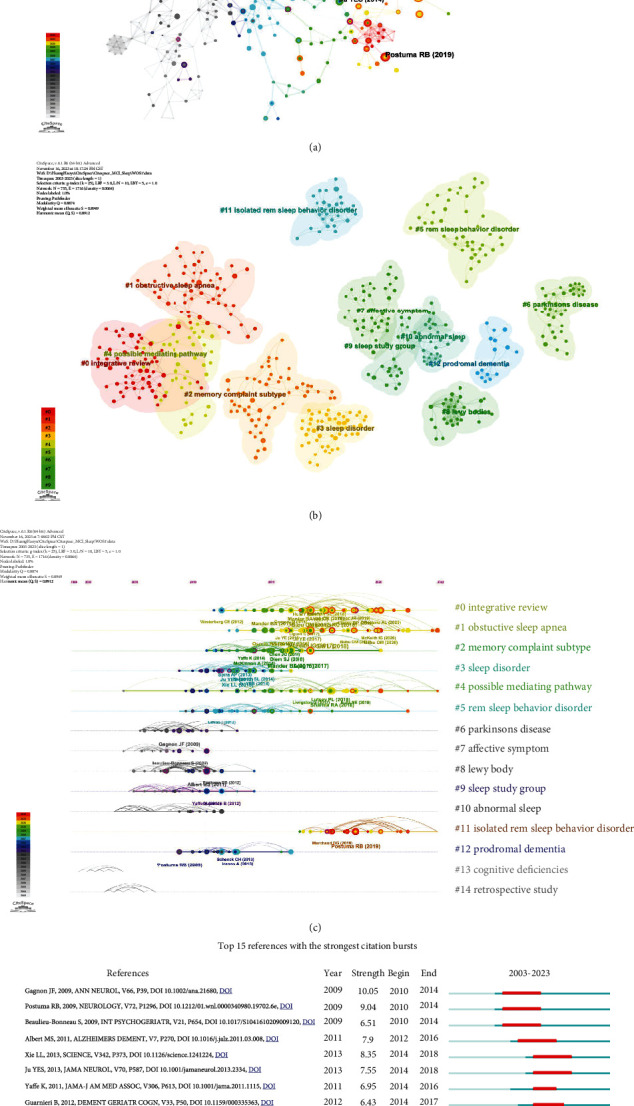
The visualization map of the cocited reference network (a). The visualization map of the cluster analysis for cocited references (b). The timeline view of clusters formed by cocited references (c). The top 15 references with the strongest citation bursts (d).

Reference cocitation analysis is a crucial tool for trend analysis and hotspot identification in specific fields.  Figure 6(a) presents the CiteSpace visual map of cocited references in the field of MCI with dyssomnias over the past two decades. Among the top ten cocited articles (refer to Table 4), there are two consensus reports, one animal experiment article, three clinical trial articles, three meta-analyses, and one review. These articles underscore the considerable attention devoted by researchers to investigating the impact of dyssomnias on cognitive disorders. The identification of dyssomnias as potential biomarkers and therapeutic targets for these cognitive disorders represents a pivotal focus in this field. Furthermore, these cited studies represent seminal publications that establish the groundwork for future research.

The cluster analysis results of cocited references are illustrated in Figure 6(b). The distinct colour blocks correspond to separate clusters in various regions.  Figure 6(c) presents the results of a cluster analysis of cocited references in the form of a timeline view. The size of the nodes corresponds to the total number of citations, while colour indicates specific time periods. The cocitations between two articles are visually represented by lines of varying colours that connect the nodes. This visualization enhances understanding of current research topics and provides insights into future directions in MCI research with dyssomnias. The graph depicts the top 15 reference clusters ranked by publication count, consisting of 735 nodes and 1716 edges. In 2022, obstructive sleep apnea (OSA) (#1) emerged as the largest active cluster with a size of 56. It was followed by possible mediating pathways (#4) with a size of 46 and isolated rapid eye movement (REM) sleep behaviour disorder (#11) with a size of 29. It is evident that significant attention has been devoted to OSA or REM behaviour disorder combined with MCI among dyssomnias. Furthermore, researchers are keen on exploring potential links between MCI and dyssomnias.

Figure 6(d) presents the top 15 references exhibiting the highest number and intensity of cited bursts, with red horizontal stripes indicating years with frequent publications and blue horizontal stripes indicating years with infrequent publications. Citation bursts refer to frequently cited references over time, and their identification can highlight hotspots within a specific time period based on reference topics. The study by Gagnon, “Mild cognitive impairment in rapid eye movement sleep behaviour disorder and Parkinson's disease”, with a burst rate of 10.05 (2010-2014), highlights the association between idiopathic REM sleep behaviour disorder and both Parkinson's disease (PD) and its idiopathic form, serving as a significant risk factor for MCI [26].

### 3.7. Analysis of Keywords

The keyword cooccurrence analysis function of CiteSpace serves as an invaluable tool for researchers to acquire a comprehensive understanding of the knowledge landscape within a specific domain by unveiling significant associations among keywords in academic literature.

**Figure 7:**
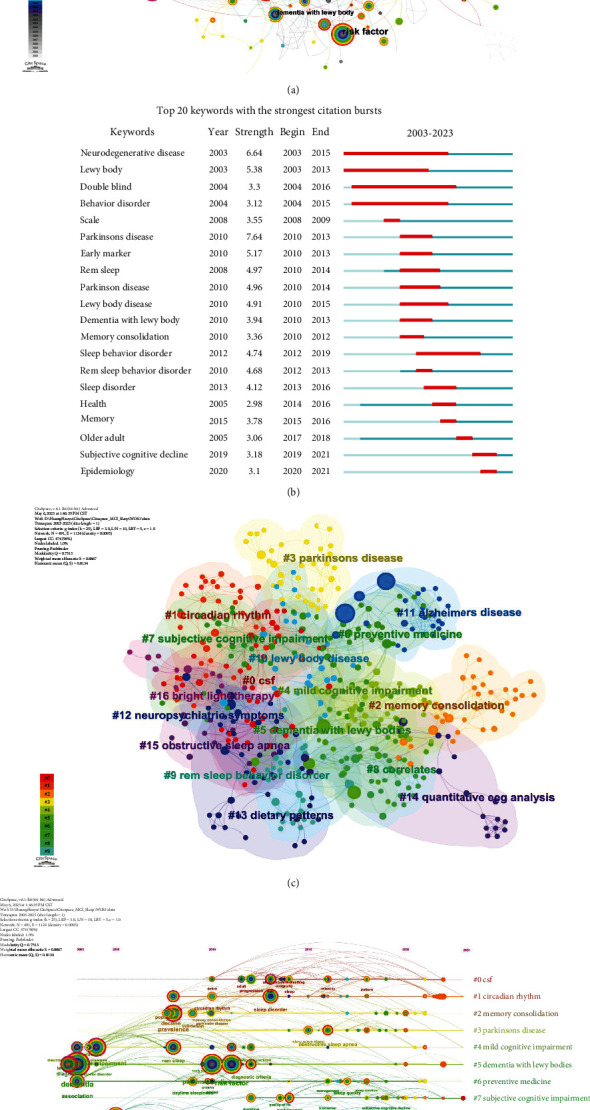
The visualization map of the keywords (a). The top 20 keywords with the strongest citation bursts (b). The visualization map of the cluster analysis for keywords (c). The timeline view of the cluster analysis for keywords (d).

The keyword cooccurrence network map displayed in Figure 7(a) was generated by CiteSpace and includes a total of 2257 keywords. The top five most frequently occurring keywords, in addition to the search term MCI, are Alzheimer's disease (AD), dementia, risk factor, and PD.  Figure 7(b) presents the top 20 keywords with the strongest citation bursts as indicated on the graph. Notably, PD, neurodegenerative disease, and Lewy body emerge as the keywords with the most intense citation bursts.

Further cluster analysis of the keywords based on their cooccurrence enables the generation of a keyword cluster map and a clustering timeline view (Figures 7(c) and 7(d)). These maps provide additional insights into the relationships and patterns among the keywords. The node at the beginning of the horizontal axis represents the initial appearance of the reference. The size of each node in the network is proportional to the number of citations for its corresponding keyword, while lines connecting nodes indicate cocited relationships. Our analysis has identified a total of 24 distinct clusters, out of which only the first 17 larger clusters are displayed by the software. These visually represented clusters are distinguished by different colours and consist of a combined total of 491 nodes and 1,124 lines. The Modularity Q value was calculated as 0.7513, indicating well-defined network clusters as it exceeds the threshold of 0.5. The mean silhouette *S* value was calculated to be 0.8867, indicating a satisfactory level of homogeneity within the clusters, surpassing the threshold of 0.5 as well [34].

During the clustering process, elements with similar characteristics were grouped together into independent clusters, each labelled to represent its contents. The resulting clusters are as follows: #0 cerebrospinal fluid (CSF), #1 circadian rhythm, #2 memory consolidation, #3 PD, #4 MCI, #5 dementia with Lewy body, #6 preventive medicine, #7 subjective cognitive impairment, #8 correlates, #9 REM sleep behaviour disorder, #10 Lewy body disease, #11 AD, #12 neuropsychiatric symptoms, #13 dietary patterns, #14 quantitative electroencephalogram (EEG) analysis, #15 OSA, and #16 bright light therapy.

## 4. Discussion

This study represents the first bibliometric analysis of research articles on MCI with dyssomnias worldwide over the past two decades, utilizing the WOSCC database, VOSviewer, and CiteSpace to explore research trends and hotspots in this field. A total of 546 articles and review papers published between 2003 and 2023 were searched and analysed.

Over the course of two decades, there has been a consistent upward trend in the annual publication count, capturing the attention of researchers in Neurosciences, Clinical Neurology, Geriatrics Gerontology, and other related fields toward exploring the association between sleep disorders and MCI. From 2003 to 2009, the growth rate exhibited a relatively gradual pace with a predominant focus on clinical research resulting in less than ten publications per year. However, following 2010, there was a notable surge in development, with a steady increase in the number of published literatures until 2018. Throughout this period, clinical research expanded, and researchers commenced investigating the intrinsic correlation and pathological mechanisms between MCI, its neuropsychiatric symptoms, and various sleep disorders. Since 2019, there has been a substantial upswing in the growth rate of literature within this field, accompanied by several high-impact meta-analyses and systematic reviews. The spectrum of neurodegenerative disorders associated with MCI has also expanded. Researchers are currently investigating effective strategies for early prevention, screening, and treatment of MCI accompanied by dyssomnias, including interventions such as bright light therapy and dietary patterns.

Among the 227 countries analysed in this study, the United States has emerged as the foremost contributor in terms of both publications (*n* = 161) and citations (*n* = 5138), underscoring its significant impact on research related to MCI with dyssomnias over the past two decades. Among the top 10 most productive institutions, six are situated in North America, two in Europe, one in Oceania, and one in Asia. This observation implies that economically advanced regions have made greater advancements in research related to MCI with dyssomnias.

In terms of individual authors, Naismith Sharon L (*n* = 23), Gagnon Jean-Francois (*n* = 17), and Postuma Ronald B (*n* = 15) emerged as the top three contributors based on publication count. Notably, an article authored by Naismith et al. from Australia [35], was the first to establish a link between objectively measured sleep disturbances and cognitive function in elderly patients with MCI. This study also investigated various subtypes of MCI with psychiatric, vascular, and neurological features. Additionally, a significant impact was observed from a large multicentre prospective cohort study published in Brain [16], which involved the collaboration of Gagnon Jean-Francois and Postuma Ronald B from Canada and received a relatively high number of citations (*n* = 402). The study confirmed the heightened risk of developing PD, dementia with Lewy bodies, and multiple system atrophy in individuals with idiopathic REM sleep behaviour disorder (iRBD). Furthermore, it demonstrated a significant increase in the conversion rate from iRBD to overt neurodegenerative syndrome due to MCI. These findings have substantial implications for potential prevention and early treatment of synucleinopathies associated with neurodegeneration.

The *Journal of Alzheimer's Disease* and *Sleep Medicine* are distinguished by their high publication volume and citation rates, respectively. *Neurology* is recognized as the most frequently cocited journal based on citations. In recent years, these journals have devoted significant attention to investigating the neural correlations and pathological mechanisms underlying the relationship between MCI and abnormal sleep patterns. Their research is focused on developing strategies for the prevention and treatment of MCI in its early stages, with the aim of halting cognitive decline and mitigating neuropsychiatric symptoms. For instance, a clinical study published in the *Journal of Alzheimer's Disease* compared patients diagnosed with iRBD and MCI (MCI-RBD) to matched patients diagnosed with MCI caused by AD (MCI-AD) [36]. This study has identified distinct neuropsychological and brain metabolic characteristics between MCI-RBD and MCI-AD, which can facilitate the diagnosis and prediction of MCI conversion to dementia. A recent review in *Sleep Medicine* has highlighted significant micro- and macrostructural changes in sleep patterns associated with aging [37], discussing their impact on cognitive function, quality of life, and the brain. Altered sleep patterns are recognized as fundamental components of both MCI and AD, with abnormal sleep accelerating the progression of AD and promoting the buildup of amyloid-*β* (A*β*) and phosphorylated tau. The article also explores potential therapeutic strategies for addressing sleep disruption, emphasizing the urgent need for testing new interventions. Moreover, Neurology articles in this research field predominantly concentrate on exploring the intrinsic pathological features of MCI with dyssomnias at a neurobiological level. Notably, a prospective cohort study investigating patients with iRBD demonstrated that MCI in individuals with iRBD was linked to functional and metabolic alterations in specific brain regions [38]. Hypometabolism within these regions served as a predictive factor for the phenotypic transition to Parkinson's disease or dementia with Lewy bodies.

Reference cocitation analysis, a bibliometric approach utilized to explore the interrelationships among articles based on their shared references, reveals the frequency of joint citations by other publications for any given pair of articles. Notably, among the top ten highly cited articles, the inaugural publication in *Sleep Medicine Reviews* [27] stands out prominently. These findings imply that sleep disturbances may serve as an indicative factor for an elevated risk of developing dementia. Insomnia was found to be specifically associated with incident AD, while sleep-disordered breathing was identified as a risk factor for all-cause dementia, AD, and vascular dementia. This article has garnered significant attention due to its ability to establish a clear link between sleep disorders and dementia, both of which are important health concerns among older adults. The insights provided can aid in identifying individuals at risk for developing dementia and optimizing early prevention strategies.

The analysis of overlay maps provides a visually intuitive approach to explore the intellectual structure, interdisciplinary connections, and evolution of research topics within a field. By examining the cooccurrence of keywords across articles, it is possible to identify areas of overlap between different disciplines and track the flow of knowledge. In the dual-map overlay of journals related to MCI with dyssomnia research, the majority of citing publications are found in the fields of Molecular Biology, Immunology, Neurology, Sports, and Ophthalmology. Meanwhile, cited articles primarily originate from the fields of Molecular Biology, Genetics, Psychology, Education, and Social Sciences.

The CiteSpace software employs keyword cluster analysis to group closely related keywords based on patterns of literature cooccurrence, thereby facilitating the identification of shared research themes. The timeline view provides a visual representation of cluster evolution, enabling researchers to track the emergence, popularity, decline, and transformation of research topics over time. The solid line on the timeline represents the duration of clustering research, while the dashed line indicates a lack of relevant literature during that period. The significance of a cluster domain increases with the inclusion of more literature. Analysing the keyword clustering timeline view (Figure 7(d)) based on research time and document count reveals a total of 8 clusters (#1, #3, #4, #5, #7, #10, #11, and #13) with a research span extending until 2023. These 8 clusters consist of an average of 140 documents each, surpassing the overall mean for all clusters (86 documents). Therefore, it is predicted that these eight clusters will remain active throughout 2023 and possess a certain degree of influence. Cluster #1, centered on circadian rhythm, encompasses keywords such as sleep disorders and diseases. Studies have indicated that disruptions in daytime activity, sleep patterns, and circadian rhythm indicators may serve as predictors for the onset of MCI or AD [39, 40]. Cluster #3, which focuses on PD, includes keywords such as OSA and slow wave sleep. Research has suggested that enhancing the quality of sleep, particularly slow wave sleep, may improve cognitive abilities [41]. Additionally, OSA is linked to an increased risk of MCI, AD, and PD [42]. Cluster #4, centered on MCI, encompasses keywords such as REM sleep, neurodegenerative disease, and cognitive impairment. A noteworthy study within this cluster is Boeve et al.'s research published in *Sleep Medicine* [17], which further corroborates the significant association between REM sleep behaviour disorder and synucleinopathy. Cluster #5, known as dementia with Lewy body, encompasses keywords such as Dementia, risk factor, and PD. A significant clinical study within this cluster revealed comparable rates of sleep disorders in AD and MCI patients. Sleep-disordered breathing is more prevalent in vascular dementia while REM behaviour disorder is frequently observed in Lewy bodies or PD dementia [23]. The seventh cluster, namely, subjective cognitive impairment (SCI), encompasses keywords such as performance, duration, and quality of life. Existing research indicates that individuals with sleep disorders often perceive subjective cognitive impairment despite having similar objective cognitive levels [43]. Sleep disorders are prevalent among the elderly population and have been associated with an increased risk of future MCI and dementia. Sleep disturbances are frequently observed in individuals with subjective cognitive impairment, highlighting the importance of sleep screening and improving sleep quality. Cluster #10, associated with Lewy body disease, is linked to the primary keyword “sleep behaviour disorder”. Clinical studies have compared neuropsychiatric symptoms (NPS) in patients with MCI-Lewy body (MCI-LB) and those with MCI-AD. The results indicate a higher prevalence and severity of NPS, visual hallucinations, and RBD in patients with MCI-LB [44]. Cognitive, parkinsonian, neuropsychiatric, sleep, and autonomic symptoms were significantly more common in patients with MCI-LB than those with MCI-AD [45]. Cluster #11 AD is focused on the keyword “sleep disturbance”. A systematic review has investigated the association between sleep disorders and APOE *ε*4 status in individuals with MCI and AD [46]. The results suggest that APOE *ε*4 is associated with poorer sleep quality, including factors such as total sleep time, REM sleep, sleep efficiency, latency, and wakefulness after sleep onset. Other studies have demonstrated age-related disruptions in sleep patterns among individuals with MCI and AD, which can contribute to cognitive decline irrespective of underlying pathology. Sleep parameters commonly affected include REM sleep, sleep efficiency, latency, and duration [10], thereby emphasizing the association between sleep disturbances and cognitive impairment. Cluster #13, dietary patterns, focuses on the concept of “quality”. This section delves into the impact of dietary patterns on sleep and cognitive performance. The Mediterranean diet has been linked to favourable effects on inflammation, oxidative stress, metabolic disorders, aging-related decline in cognitive function, and overall health. Adherence to this dietary regimen is associated with adequate sleep duration and enhanced sleep quality [47]. Furthermore, an additional study underscores the significance of incorporating dietary and sleep interventions as preventive measures against cognitive decline [48].

However, it is important to acknowledge the limitations of this study. Firstly, the accuracy and reliability of bibliometric analysis are contingent upon data quality and sources; as such, our reliance solely on the WoSCC database may have resulted in relevant studies being overlooked. Secondly, our restriction to English-language articles introduces a potential language bias that may exclude valuable research conducted in other languages. Therefore, further investigation across multiple databases and languages is necessary.

## 5. Conclusions

By utilizing CiteSpace, VOSviewer, and WOS analysis tools, we have gained valuable insights into the advancements, emerging areas, and future directions of research in the field of MCI with dyssomnias. It is crucial that future research continues to prioritize exploring the neural mechanisms underlying the intricate relationship between MCI and dyssomnias. Moreover, the development of more efficacious intervention programs targeting dyssomnias in individuals with MCI is imperative. Continued investigation in these areas will not only enhance our understanding of this overlapping domain but also pave the way for potential therapeutic interventions.

## Figures and Tables

**Table 1:** The top 10 most cited articles.

Rank	First author	Title	Journal	Cited	Highlight	References
1	Ronald B Postuma	Risk and Predictors of Dementia and Parkinsonism in Idiopathic REM Sleep Behaviour Disorder: A Multicentre Study	*Brain*	402	The study has confirmed a high risk of phenoconversion to overt neurodegenerative disease in individuals with rapid eye movement sleep behaviour disorder (RBD) and has identified several predictors of phenoconversion.	[16]
2	Bradley F Boeve	Clinicopathologic Correlations in 172 Cases of Rapid Eye Movement Sleep Behavior Disorder with or without a Coexisting Neurologic Disorder	*Sleep Medicine*	225	The study demonstrates that RBD typically manifests before cognitive impairment, parkinsonism, or autonomic dysfunction in individuals with an underlying neurodegenerative disorder. These findings provide strong evidence for the association between RBD and synucleinopathies.	[17]
3	Bradley F Boeve	Melatonin for Treatment of REM Sleep Behavior Disorder in Neurologic Disorders: Results in 14 Patients	*Sleep Medicine*	225	The findings of this study suggest that melatonin may be a viable option as either a standalone or adjunctive therapy for patients with RBD who present various neurological symptoms and disorders.	[18]
4	Yonas E Geda	Neuropsychiatric Symptoms in Alzheimer's Disease: Past Progress and Anticipation of the Future	*Alzheimers & Dementia*	224	The study proposes four potential mechanisms that establish a connection between neuropsychiatric symptoms (including depression, apathy, sleep disturbances, agitation, and psychosis) and MCI or Alzheimer's disease dementia.	[19]
5	Jean Woo	Frailty Screening in the Community Using the FRAIL Scale	*Journal of The American Medical Directors Association*	216	Among individuals aged 65 years and older who are frail or prefrail, approximately 60% exhibit MCI along with suboptimal sleep quality. Additionally, they consume a higher number of medications, especially sleeping pills.	[20]
6	Ronald B Postuma	Rapid Eye Movement Sleep Behavior Disorder and Risk of Dementia in Parkinson's Disease: A Prospective Study	*Movement Disorders*	205	All participants enrolled in the study exhibited MCI; the findings revealed that a loss of baseline REM sleep atonia predicted subsequent dementia development, as well as the emergence of hallucinations and cognitive fluctuations.	[21]
7	Carmen E Westerberg	Concurrent Impairments in Sleep and Memory in Amnestic Mild Cognitive Impairment	*Journal of The International Neuropsychological Society*	183	The findings suggest that sleep disturbances in individuals with amnestic MCI contribute to memory deficits by disrupting the consolidation of memories dependent on sleep.	[22]
8	Biancamaria Guarnieri	Prevalence of Sleep Disturbances in Mild Cognitive Impairment and Dementing Disorders: A Multicenter Italian Clinical Cross-Sectional Study on 431 Patients	*Dementia and Geriatric Cognitive Disorders*	183	More than 60% of individuals experienced one or more sleep disturbances, often occurring in conjunction with each other without any discernible pattern of cooccurrence. The frequency of any sleep disorder was similar between those with Alzheimer's disease and MCI.	[23]
9	Bradley F Boeve	Validation of the Mayo Sleep Questionnaire to Screen for REM Sleep Behavior Disorder in an Aging and Dementia Cohort	*Sleep Medicine*	181	The study suggests that the Mayo Sleep Questionnaire demonstrates sufficient sensitivity and specificity to diagnose RBD in elderly individuals with cognitive impairment and/or parkinsonism.	[24]
10	Martijn L. T. M. Müller	Cholinergic Dysfunction in Parkinson's Disease	*Current Neurology and Neuroscience Reports*	159	Given that RBD serves as an antecedent marker for cognitive impairment and dementia in Parkinson's Disease, these findings suggest the possibility of early degeneration in the cholinergic system in this condition.	[25]

**Table 2:** The top 10 Countries/territories with the highest number of publications.

Rank	Countries/territories	First year	Record count	Centrality	Citations
1	USA	2003	161	0.07	5138
2	China	2013	100	0.00	1184
3	Italy	2008	75	0.00	2130
4	Canada	2009	55	0.09	2490
5	England	2010	48	0.37	1606
6	Australia	2010	42	0.13	1809
7	Spain	2005	34	0.16	2139
8	South Korea	2011	30	0.00	725
9	Germany	2011	29	0.17	1073
10	France	2004	27	0.05	1473

**Table 3:** The top 10 cited and cocited journals with the highest publication and citation volumes.

Rank	Cited journals	Documents	Citations	Cocited journals	Documents	Citations
1	*Journal of Alzheimers Disease*	44	731	*Neurology*	426	1715
2	*Sleep Medicine*	25	1233	*Sleep*	338	1520
3	*Sleep*	22	639	*Movement Disorders*	164	901
4	*Frontiers in Aging Neuroscience*	21	384	*Sleep Medicine*	263	654
5	*Frontiers in Neurology*	13	117	*Journal of Alzheimer's Disease*	256	643
6	*International Journal of Geriatric Psychiatry*	13	447	*Alzheimers & Dementia*	255	599
7	*International Psychogeriatrics*	11	256	*Journal of the American Geriatrics Society*	288	559
8	*Parkinsonism & Related Disorders*	10	352	*Neurobiology of Aging*	217	512
9	*Journal of Geriatric Psychiatry and Neurology*	9	213	*Brain*	230	474
10	*Journal of Sleep Research*	9	194	*Annals of Neurology*	232	441

**Table 4:** The top 10 cocited references.

Rank	Count	First author	Year	Title	References
1	34	Le Shi	2018	Sleep Disturbances Increase the Risk of Dementia: A Systematic Review and Meta-Analysis	[27]
2	33	Yue Leng	2017	Association of Sleep-Disordered Breathing With Cognitive Function and Risk of Cognitive Impairment: A Systematic Review and Meta-Analysis	[28]
3	31	Bryce A Mander	2016	Sleep: A Novel Mechanistic Pathway, Biomarker, and Treatment Target in the Pathology of Alzheimer's Disease?	[29]
4	29	Ian G McKeith	2017	Diagnosis and Management of Dementia with Lewy Bodies: Fourth Consensus Report of the DLB Consortium	[30]
5	26	Omonigho M Bubu	2017	Sleep, Cognitive Impairment, and Alzheimer's Disease: A Systematic Review and Meta-Analysis	[10]
6	26	Ronald C Petersen	2018	Practice Guideline Update Summary: Mild Cognitive Impairment: Report of the Guideline Development, Dissemination, and Implementation Subcommittee of the American Academy of Neurology	[31]
7	24	Ronald B Postuma	2019	Risk and Predictors of Dementia and Parkinsonism in Idiopathic REM Sleep Behaviour Disorder: A Multicentre Study	[16]
8	22	Ram A Sharma	2018	Obstructive Sleep Apnea Severity Affects Amyloid Burden in Cognitively Normal Elderly. A Longitudinal Study	[32]
9	21	Lulu Xie	2013	Sleep Drives Metabolite Clearance from the Adult Brain	[33]
10	20	Jean-François Gagnon	2009	Mild Cognitive Impairment in Rapid Eye Movement Sleep Behavior Disorder and Parkinson's Disease	[26]

## Data Availability

The bibliometric data supporting the findings of this study are available upon request from the corresponding author, in accordance with established academic practices.
